# Protein L: a novel reagent for the detection of Chimeric Antigen Receptor (CAR) expression by flow cytometry

**DOI:** 10.1186/1479-5876-10-29

**Published:** 2012-02-13

**Authors:** Zhili Zheng, Nachimuthu Chinnasamy, Richard A Morgan

**Affiliations:** 1 Surgery Branch, Center for Cancer Research, National Cancer Institute, National Institutes of Health, Bethesda, MD 20892, USA

## Abstract

**Background:**

There has been significant progress in the last two decades on the design of chimeric antigen receptors (CAR) for adoptive immunotherapy targeting tumor-associated antigens. Structurally CARs consist of a single chain antibody fragment directed against a tumor-associated antigen fused to an extracellular spacer and transmembrane domain followed by T cell cytoplasmic signaling moieties. Currently several clinical trials are underway using gene modified peripheral blood lymphocytes (PBL) with CARs directed against a variety of tumor associated antigens. Despite the improvements in the design of CARs and expansion of the number of target antigens, there is no universal flow cytometric method available to detect the expression of CARs on the surface of transduced lymphocytes.

**Methods:**

Currently anti-fragment antigen binding (Fab) conjugates are most widely used to determine the expression of CARs on gene-modified lymphocytes by flow cytometry. The limitations of these reagents are that many of them are not commercially available, generally they are polyclonal antibodies and often the results are inconsistent. In an effort to develop a simple universal flow cytometric method to detect the expression of CARs, we employed protein L to determine the expression of CARs on transduced lymphocytes. Protein L is an immunoglobulin (Ig)-binding protein that binds to the variable light chains (kappa chain) of Ig without interfering with antigen binding site. Protein L binds to most classes of Ig and also binds to single-chain antibody fragments (scFv) and Fab fragments.

**Results:**

We used CARs derived from both human and murine antibodies to validate this novel protein L based flow cytometric method and the results correlated well with other established methods. Activated human PBLs were transduced with retroviral vectors expressing two human antibody based CARs (anti-EGFRvIII, and anti-VEGFR2), two murine antibody derived CARs (anti-CSPG4, and anti-CD19), and two humanized mouse antibody based CARs (anti-ERBB2, and anti-PSCA). Transduced cells were stained first with biotin labeled protein L followed by phycoerythrin (PE)-conjugated streptavidin (SA) and analyzed by flow cytometry. For comparison, cells were stained in parallel with biotin conjugated goat-anti-mouse Fab or CAR specific fusion proteins. Using protein L, all CAR transduced lymphocytes exhibited specific staining pattern ranging from 40 to 80% of positive cells (compared to untransduced cells) and staining was comparable to the pattern observed with anti-Fab antibodies.

**Conclusion:**

Our data demonstrate the feasibility of employing Protein L as a general reagent for the detection of CAR expression on transduced lymphocytes by flow cytometry.

## Background

**Figure 1:**
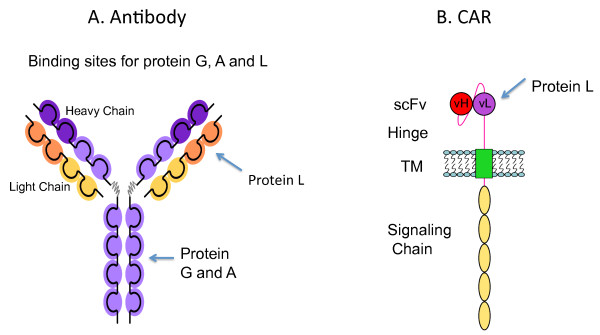
**A. Illustration showing the binding sites (arrows) of Protein A, Protein G, and Protein L to the heavy and light chain regions of the antibody**. Protein L binding is restricted to those antibodies that contain kappa light chains. B. Schema showing Protein L binding to the kappa light chain of a single chain variable fragment (scFv) portion of a chimeric antigen receptor (CAR). TM, transmembrane region of CAR; vH, variable heavy chain; vL, variable light chain.

Adoptive immunotherapy using T lymphocytes genetically modified to express a chimeric antigen receptor (CAR) combines the beneficial effects of both antibody and T-cell mediated immune responses. Typically CARs consists of a single chain antibody fragment (scFv) directed against tumor associated cell surface antigen fused to extracellular spacer and transmembrane domains followed by various combination of cytoplasmic signaling moieties such as CD3 zeta, CD28, OX40 or 4-1BB (Figure 1B). Currently a number of early phase clinical trials are underway using gene-modified peripheral blood lymphocytes (PBL) with CARs directed against a variety of tumor antigens [1]. Since the first CAR was reported in 1989 [2], there have been significant improvements in the design of CAR for optimal antigen recognition, enhanced T cell function and survival *in vivo *[3]. The number of target antigens that have been shown to be suitable for CAR based therapies is steadily expanding, indicating the potential promise of this approach in tumor immunotherapy [3,4]. Despite the advancements in the design of CARs and expansion of number of target antigens, there is no universal flow cytometric method available to detect the expression of CARs on the surface of lymphocytes.

To determine the level of expression of CARs on gene modified lymphocytes by flow cytometry, T cells have to be stained with specific ligands or antibodies conjugated with fluorochromes. For example, anti-ERBB2 and anti-VEGFR2 specific CAR expression is detected by ERBB2-fragment crystallizable (Fc) or VEGFR2-Fc fusion proteins, respectively, followed by fluorochrome conjugated anti-human IgG Fc antibody [5,6]. In other cases anti-human IgG-Fab; or anti-mouse IgG-Fab antibodies are used in flow cytometric analysis [7,8]. However, variations between polyclonal antibody preparations often lead to inconsistent results. Furthermore, staining with many different kinds of antibodies can be time consuming and labor intensive. Given the limitations of existing approaches the need for a reliable and simple method for the detection of CAR expression by flow cytometry on lymphocytes is evident. To address this issue, we employed a protein L based assay as a general method to determine the expression of various types of CARs on transduced lymphocytes.

Protein L is a bacterial surface protein isolated from *Peptostreptoccocus magnus *that selectively binds to variable light chains (kappa chain) of immunoglobulin without interfering with antigen binding property of the antibodies (Abs) [9,10]. Protein L specifically binds Ig through light chain interaction, from which the name was suggested (Figure 1A). It consists of 719 amino acid residues with a molecular weight of 76 kD and binds to light chain of all classes of immunoglobulin such as IgG, IgM, IgA, IgE, IgD, and Fab fragments [11] unlike other antibody-binding proteins such as protein A and G which bind to Fc portion of immunoglobulin (Figure 1A). It also binds to single-chain antibody fragments (scFv) making it a convenient reagent to detect the cell surface expression of CARs.

In this study, we tested the possibility of using protein L as a general reagent to detect the expression of CARs on transduced PBLs by flow cytometry. We employed CARs constructed from human as well as murine derived scFvs to validate this new flow cytometric method. Our data presented here support the feasibility of employing protein L as a general reagent to detect the cell surface expression of multiple CARs by flow cytometry.

## Methods

### PBLs and retroviral transduction

PBLs used in the study were obtained from patients under institutional review board approved clinical protocols at the Surgery Branch, National Cancer Institute, National Institutes of Health, Bethesda, MD. All patients gave informed consent for sample procurement. PBLs were stimulated with AIM-V medium (Invitrogen, Carlsbad, CA) supplemented with 5% human AB serum (Valley Biomedicals, Winchester, VA), 300 IU/ml IL2 (Aldesleukin Proleukin^®^, Novartis, Basel, Switzerland) and 50 ng/ml OKT3 (MuromonAB-CD3, Orthoclone OKT3, Ortho Biotech, Raritan, NJ) for two days before transduction with retroviral supernatants expressing various CAR constructs. MSGV1-based retroviral vectors expressing CARs against tumor-associated antigens ERBB2, VEGFR2, CSPG4, and CD19 were previously described in detail [5-8]. Anti-EGFRvIII and PSCA CARs are based on a human and a humanized mouse antibody respectively. Vector production and PBL transduction was also previously described in detail [5,7]. Briefly, 2 ml of viral vector supernatants expressing the various CARs were diluted with an equal volume of AIM-V medium supplemented with 5% human AB serum and added into one well of 6-well plates previously coated with RetroNectin (Takara Bio Inc., Otsu, Japan). Plates were then loaded with retroviral supernatants by centrifugation at 32°C, 2000 g for 2 hours (Sorvall Legend RT, Newtown, CT). Transduction was carried out by removing the vector supernatant and adding 2 × 10^6 ^activated PBLs per well, centrifugation at 1000 g for 10 minutes and the plates were then incubated at 37°C with 5% CO_2_. The transduction procedure was repeated on the following day. Five to seven days after transduction, PBLs were used for FACS analysis using protein-L and other antibodies as described below.

### Fluorescence-activated cell sorting (FACS) staining

Biotinylated protein L was purchased from GeneScript (Piscataway, NJ) reconstituted in phosphate buffered saline (PBS) at 1 mg/ml and stored at 4°C. Biotin-SA-conjugated goat-anti-mouse IgG (Fab) used to detect murine CARs (CD19 and CSPG4) was from Jackson ImmunoResearch laboratories, Inc, (West Grove, PA), ERBB2-Fc fusion protein specific to human anti-ERBB2 CAR was obtained from R&D Systems (Minneapolis, MN). For FACS staining, 1 × 10^6 ^cells were harvested and placed into a 5 ml snap-cap tube (Becton Dickinson, Franklin Lakes, NJ), and washed three times with 3 ml of ice-cold 1 × PBS containing 4% bovine serum albumin (BSA) (BSA fraction V, Fisher Scientific, Fair Lawn, NJ) wash buffer. In order to achieve optimal staining, all possible carry-over immunoglobulin in culture media supplemented with serum should be removed from the sample by washing three times. After wash, cells were resuspended in 0.2 ml of the ice-cold wash buffer and incubated either with 1 μg of protein L, 1 μg of ERBB2-Fc or 20 μg of goat-anti-mouse Abs respectively at 4°C for 45 minutes. Cells were washed with 3 ml of the ice-cold wash buffer three times, and then incubated (in the dark) with 10 μl of phycoerythrin (PE) -conjugated streptavidin (SA-PE, 5 μg/ml) (BD Bioscience, San Jose, CA) in 0.2 ml of the wash buffer for samples with protein L and goat-anti-mouse IgG. Cells incubated with human ERBB2-Fc were stained with 10 μl PE labeled goat-anti-human Fc IgG (Jackson ImmunoResearch laboratories, Inc, West Grove, PA). Immunofluorescence staining was analyzed as the relative log fluorescence of live cells, determined using a FACscan flow cytometer (BD). A combination of forward angle light scatter and propidium iodide (PI) staining was used to gate out the dead cells, and 1 × 10^5 ^cells were analyzed. All FACS data was analyzed using FlowJo 8.1.1 software (TreeStar, Ashland, OR).

## Results and discussion

**Figure 2:**
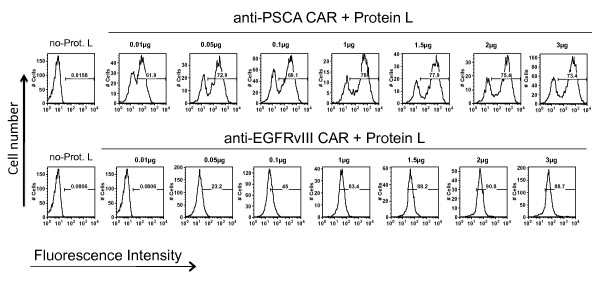
**Titration of Protein L concentration required for optimal FACS analysis. Activated PBLs were transduced with retroviral vector expressing anti-PSCA-CAR or anti-EGFRvIII-CAR and analyzed for CAR expression on day 8**. One million cells were stained with 0.01, 0.05, 0.1, 1.0, 1.5, 2.0 or 3.0 μg/sample of biotinylated Protein L. The cells were then washed and stained with phycoerythrin (PE)-conjugated streptavidin (SA). Cells were analyzed using a FACScan flow cytometer and the data analyzed with FlowJo software. Result of a representative experiment from four independent experiments is presented. No-Prot-L, samples stained with SA-PE alone.

We utilized the specific binding property of protein L to immunoglobulin light chains and scFvs to develop a general flow cytometric method for the determination of CAR expression on genetically engineered lymphocytes. Figure 1A depicts the binding of protein L on the light chains of antibodies and scFvs compared to other bacterial proteins A and G, which bind to Fc portion of the immunoglobulin. To test if protein L binds specifically on PBL transduced with CARs (Figure 1B), and to optimize the concentration of protein L needed for staining, we performed a titration experiment to determine the optimal concentration of biotinylated Protein L required for FACS analysis. PBL transduced with an anti-PSCA CAR (based on a humanized murine antibody) or an anti-EGFRvIII CAR (based on a human antibody) were used for this titration experiment. Protein L was added at 0.01, 0.05, 0.1, 1.0, 1.5, 2.0 or 3 μg for staining of 1 × 10^6 ^lymphocytes in 0.2 ml 1 × PBS containing 4% BSA. Following addition of SA-PE, CAR expressing lymphocytes exhibited a specific staining pattern (compared to control SA-PE stained cells) as measured by the flow cytometry (Figure 2). Optimal staining was observed at 1 μg protein L/sample containing 1 × 10^6 ^lymphocytes expressing either murine or human CARs. Protein L binding was highly specific to CAR expression in lymphocytes and displayed a concentration dependent binding as measured by the FACS analysis.

**Figure 3:**
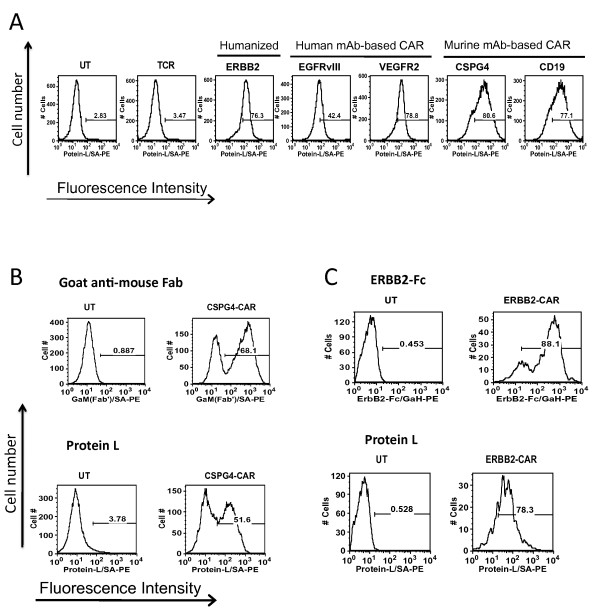
**A. FACS analysis of PBL transduced with various human or murine monoclonal antibody (mAb) based CARs**. Five days following the transduction of PBLs with retroviral vectors expressing CARs targeting ERBB2, EGFRvIII, VEGFR2, CSPG4 antigen or CD19, cells were first stained with biotinylated protein L followed by PE-conjugated streptavidin (SA). Untransduced (UT) cells and T cell receptor (TCR) transduced cells were used as negative controls. Cells were analyzed using a FACScan flow cytometer and the data analyzed with FlowJo software. **B**. Comparison of Protein L staining to other established methods of detection of CAR expression. PBL expressing murine mAb-based anti-CSPG4 CAR were analyzed for CAR expression using goat-anti-mouse fragment antigen binding (Fab) versus protein L. These results are representative of five independent determinations. **C**. Humanized mAb-based anti-ERBB2 CAR transduced PBL were analyzed for CAR expression using a CAR-specific ERBB2-fragment crystallizable (Fc) fusion protein versus protein L. The result presented here is a representative experiment of three independent determinations.

Following optimization of the concentration of biotinylated protein L required for staining of the PSCA-CAR and EGFRvIII-CAR expressing lymphocytes, we next tested general applicability of this method using lymphocytes transduced with five different CARs. Activated human PBLs were transduced with two human antibody-derived CARs directed against EGFRvIII, VEGFR2, two murine mAb-based CARs reactive with CSPG4 and CD19, and one humanized mouse antibody-based CAR recognizing ERBB2. Seven days following transduction, lymphocytes were stained with 1 μg of biotin-labeled protein L followed by incubation with PE-conjugated streptavidin and analyzed by flow cytometry. As shown in Figure 3A, CAR specific protein L staining was observed in all five PBL expressing CARs, whereas only the background staining was seen in the control cells that were either untransduced (UT) or T cell receptor (TCR) transduced PBL.

Consistent results were obtained when the experiments were repeated with CAR engineered PBL from different donors (a total of four donors were analyzed, data not shown). We have found protein L to be an extremely useful reagent for the analysis of CAR-engineered cells, and have reliably used protein L to detected CAR expression with 6 out of 6 different CAR vectors. While Protein L effectively binds most subtypes of kappa light chains (with the exception of VκII), it does not bind to lambda (λ) light chains, but these account for only a minority of antibodies (Nilson, B.H., et al. 1992, 1993).

In order to further validate the reliability of protein L as a suitable reagent to determine the expression of CARs on PBL, protein L staining was compared with two established methods (Figure 3B and 3C). As shown in Figure 3B, the percentage of CAR positive PBL determined by protein L staining (52%) was in close agreement with experimental data obtained from goat anti-mouse Fab staining used to determine the percentage of CSPG4 CAR expressing cells (68%). In another experiment lymphocytes expressing an ERBB2-CAR were stained either with protein L or ERBB2-Fc/goat-anti-human IgG. A comparable staining pattern was again observed in FACS analysis (78% vs. 88% for protein L and the ERBB2-Fc respectively). To determine the intra-assay variation, the experiments were repeated with samples from different donors and comparable results obtained (data not shown). These results suggest that CAR expression in PBL could be reliably detected using a protein L based FACS assay.

## Conclusion

In this study, we established the suitability of protein L staining as a novel and general reagent for the determination of CAR expression in PBL. Furthermore, staining is rapid and uses fewer reagents than comparable protocols. The protein L based FACS assay described in this study allowed us to readily determine the expression of CARs on transduced PBL using six unrelated CAR constructs of human, mouse, and chimeric origin. This method could be a useful tool for the quantification of nearly any CAR expressed in transduced a lymphocyte population during preclinical development, and potentially in clinical samples from cancer patients treated with lymphocytes engineered to express CARs in human gene therapy clinical trials.

## Competing interests

The authors declare that they have no competing interests.

## Authors' contributions

ZZ, performed the vector transductions and the FACS analysis of the data, and drafted the manuscript. NC, participated in its design and coordination and helped to draft the manuscript. RAM, designed the experiments, helped analyze the data, and wrote the draft manuscript. All authors read and approved the final manuscript.
